# Supplementary material for: The Construction and Exploration of a Comprehensive MicroRNA Centered Regulatory Network in Foxtail Millet (Setaria italica L.)
Source: Front Plant Sci. 2022 May 6;13:848474. doi: 10.3389/fpls.2022.848474 (PMC9121102; doi:10.3389/fpls.2022.848474)
Supplement: Supplementary file 6 [file Data_Sheet_6.zip › Sit-miRNovel8_Seita.1G177900.1_1897_TPlot.pdf]

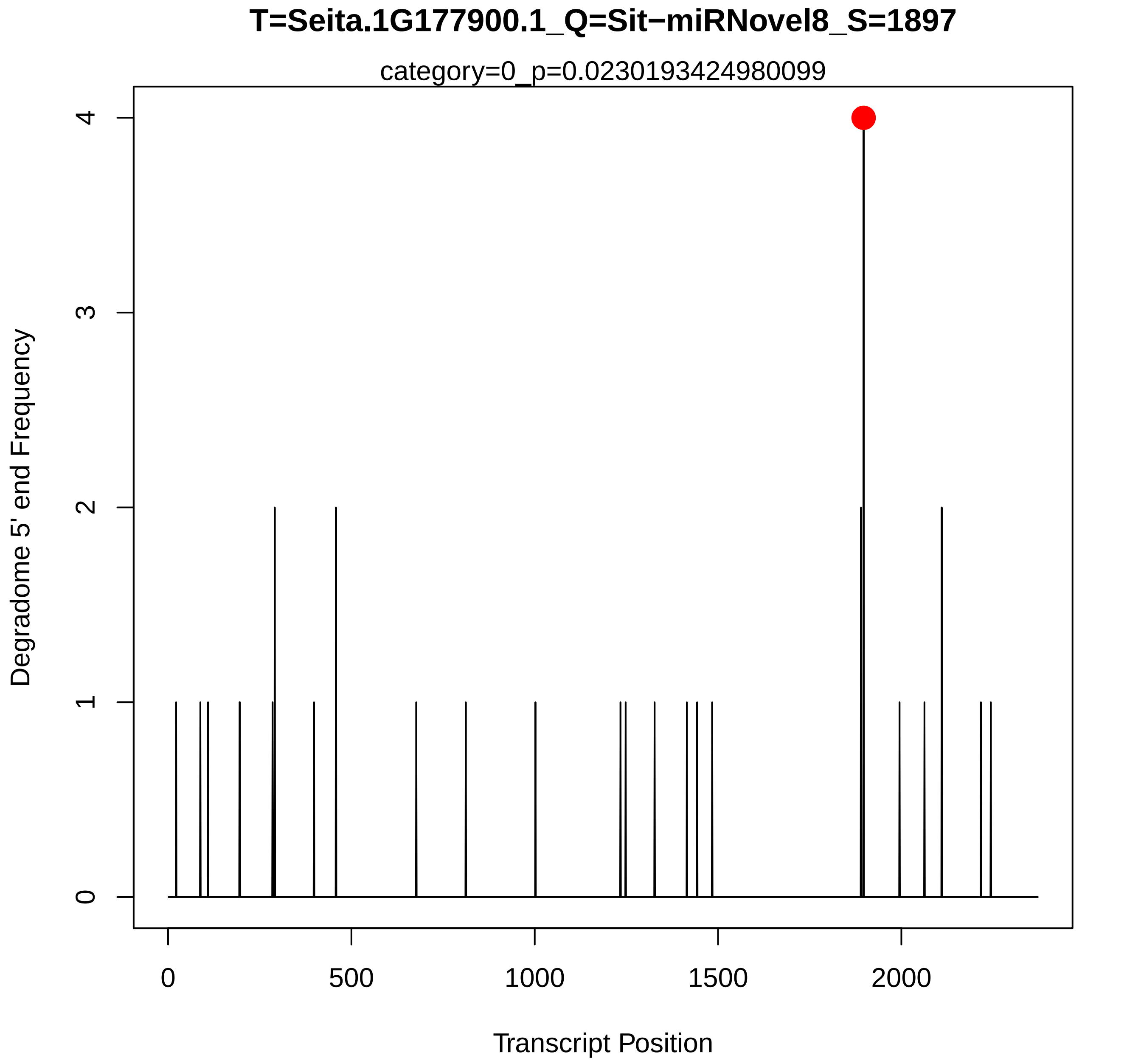

T=Seita.1G177900.1_Q=Sit−miRNovel8_S=1897
category=0_p=0.0230193424980099
Degradome 5' end Frequency
0
1
2
3
4
0
500
1000
1500
2000
Transcript Position
